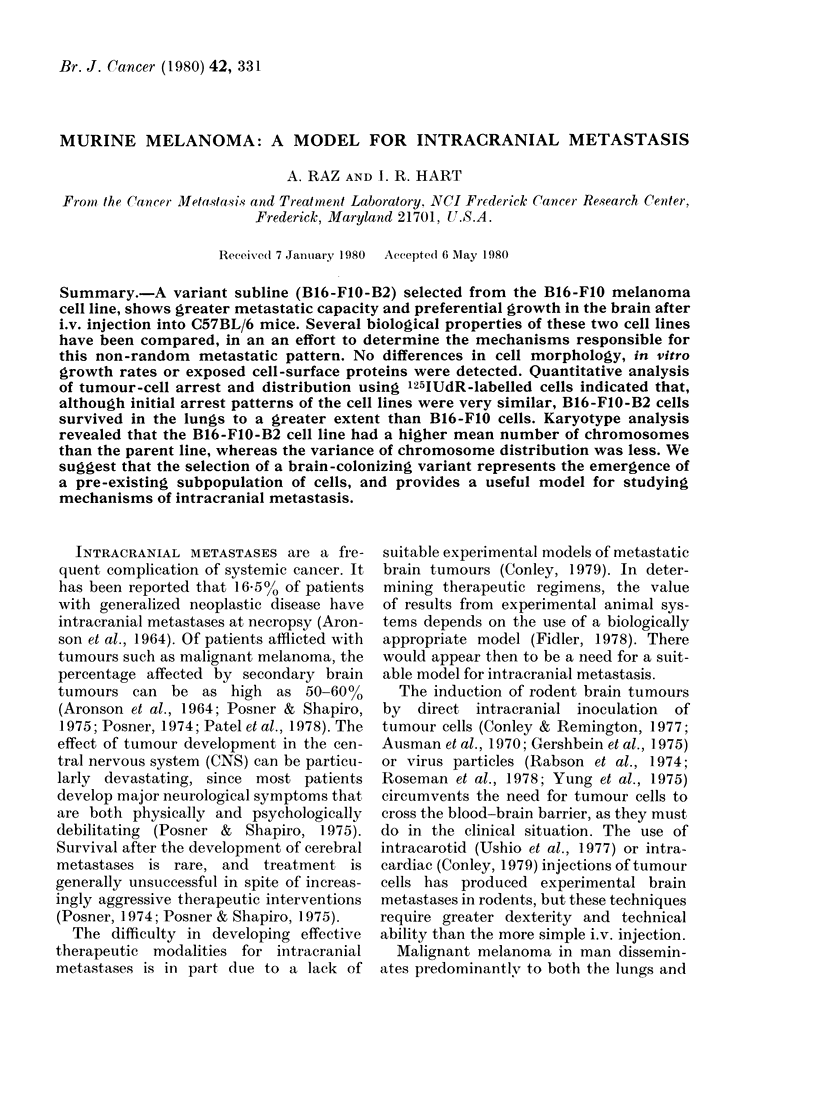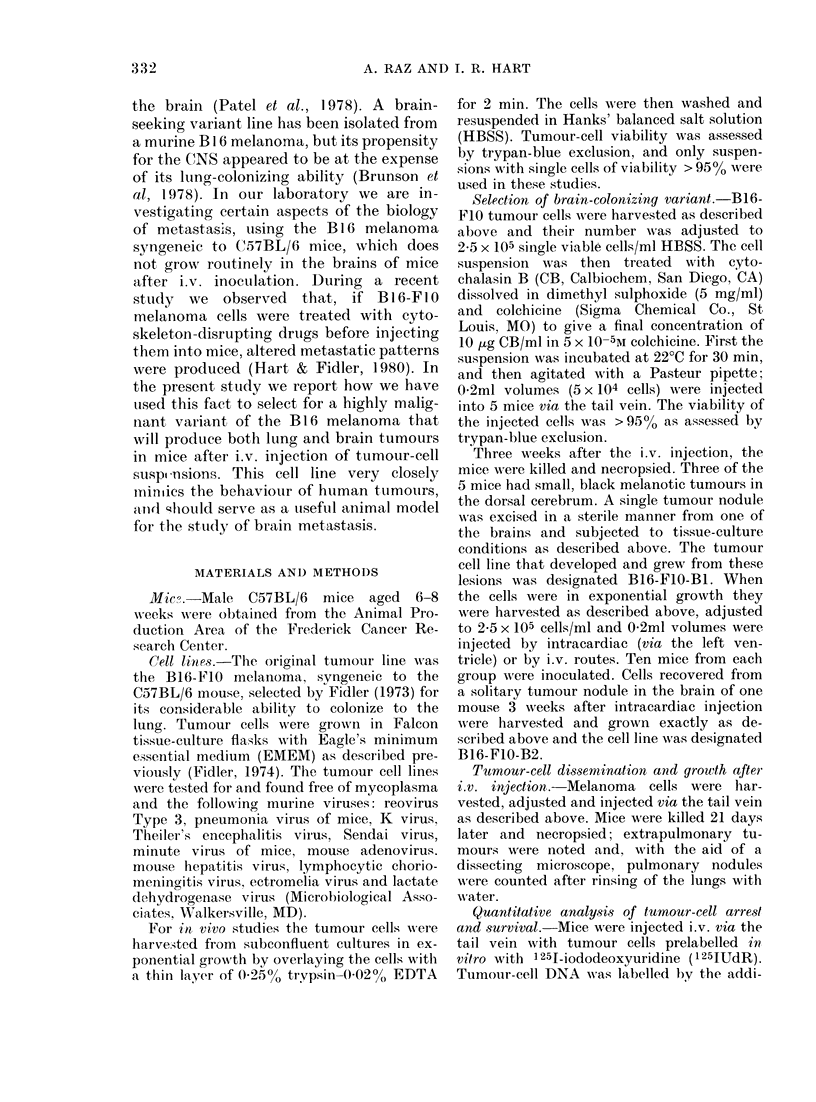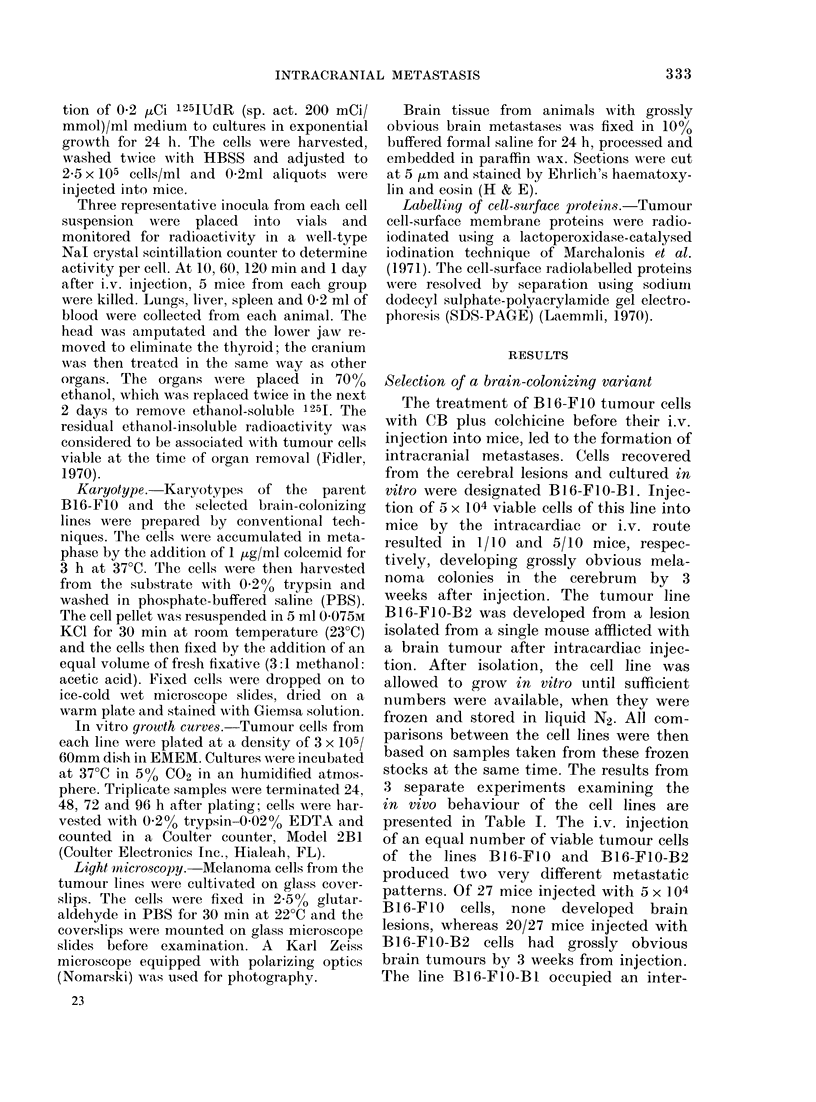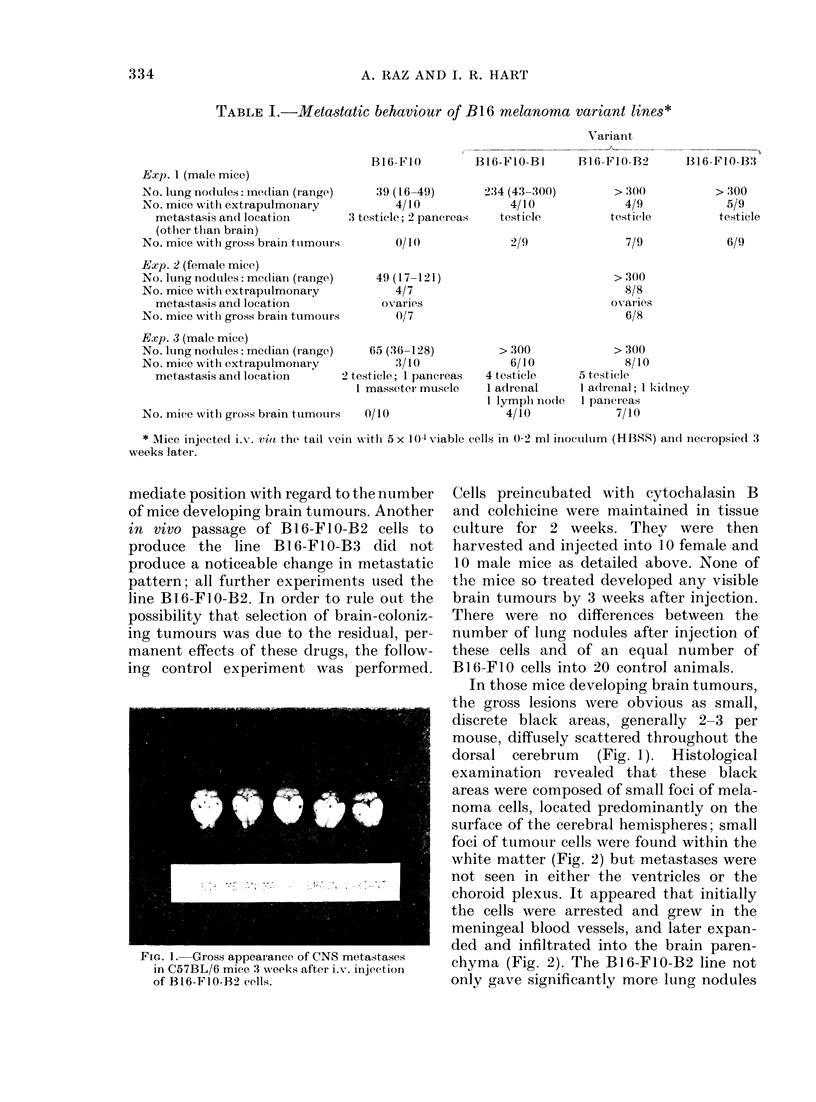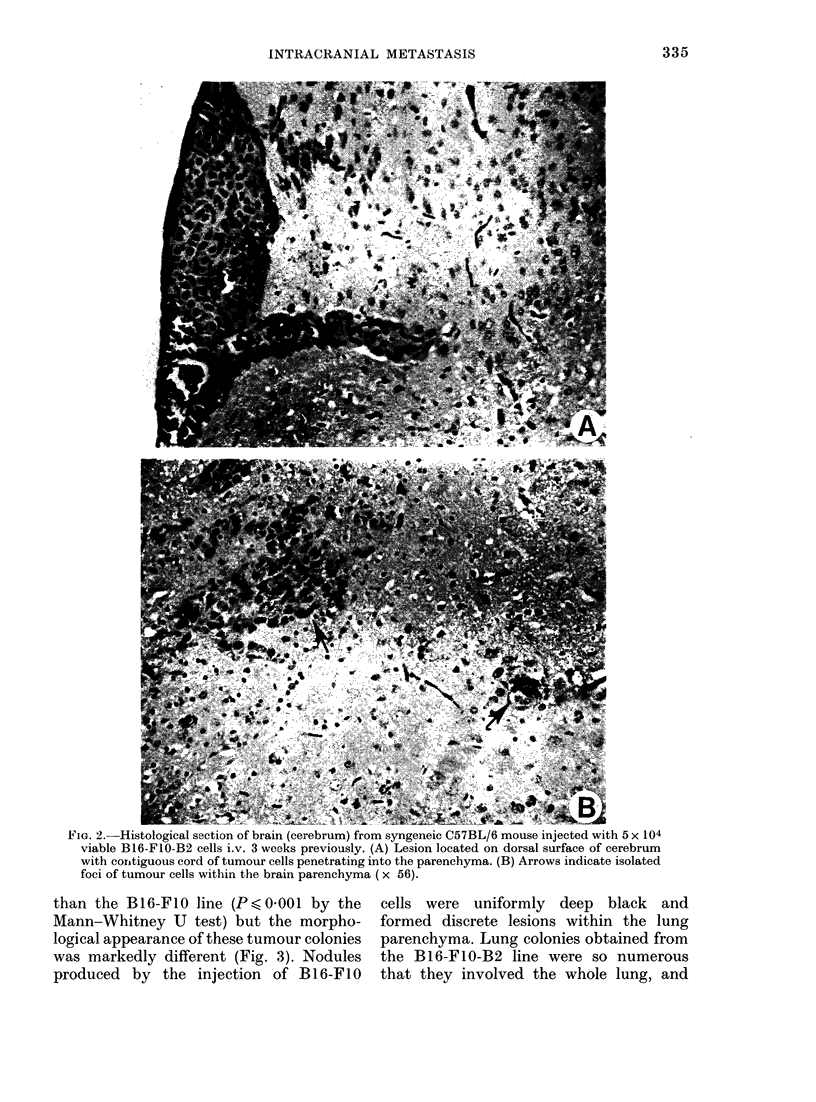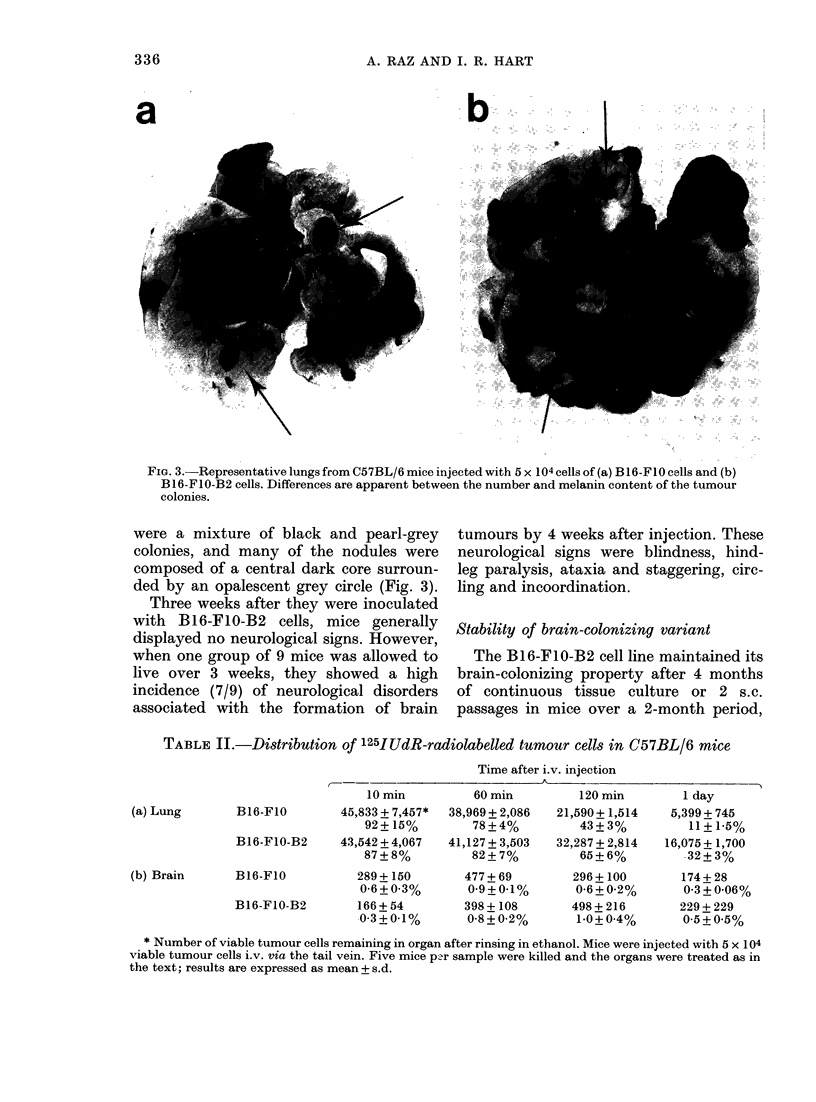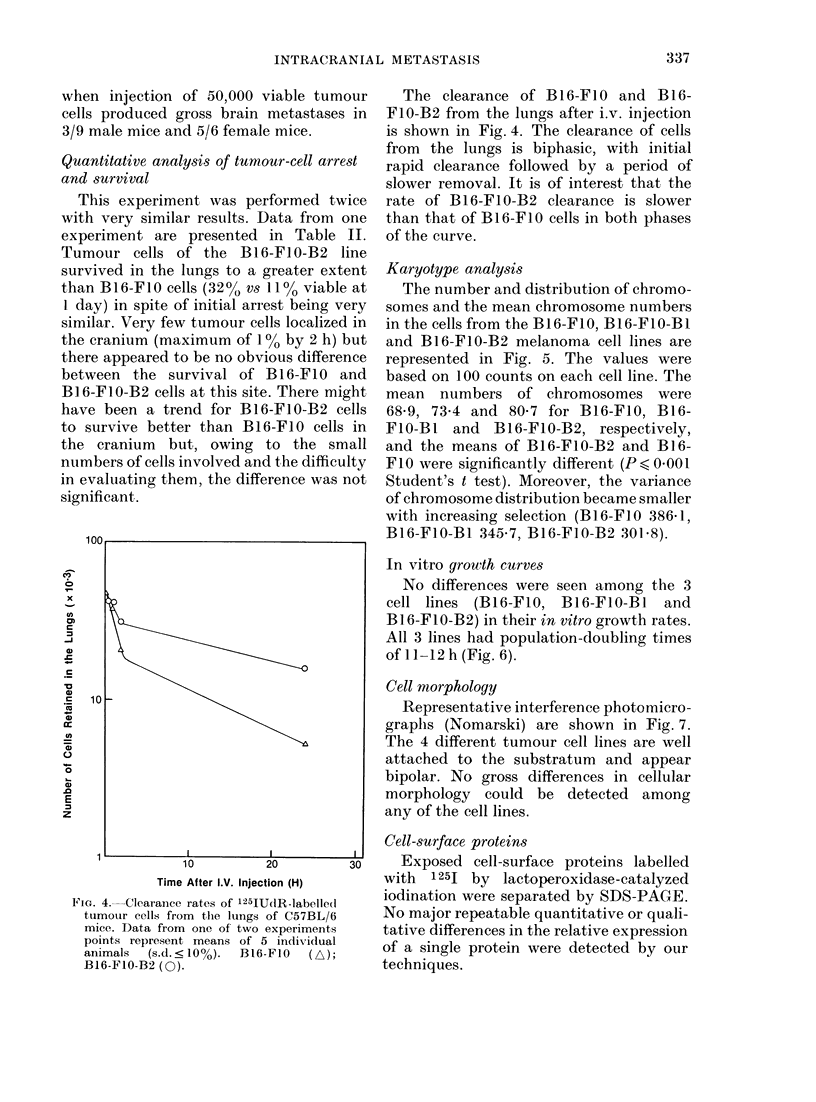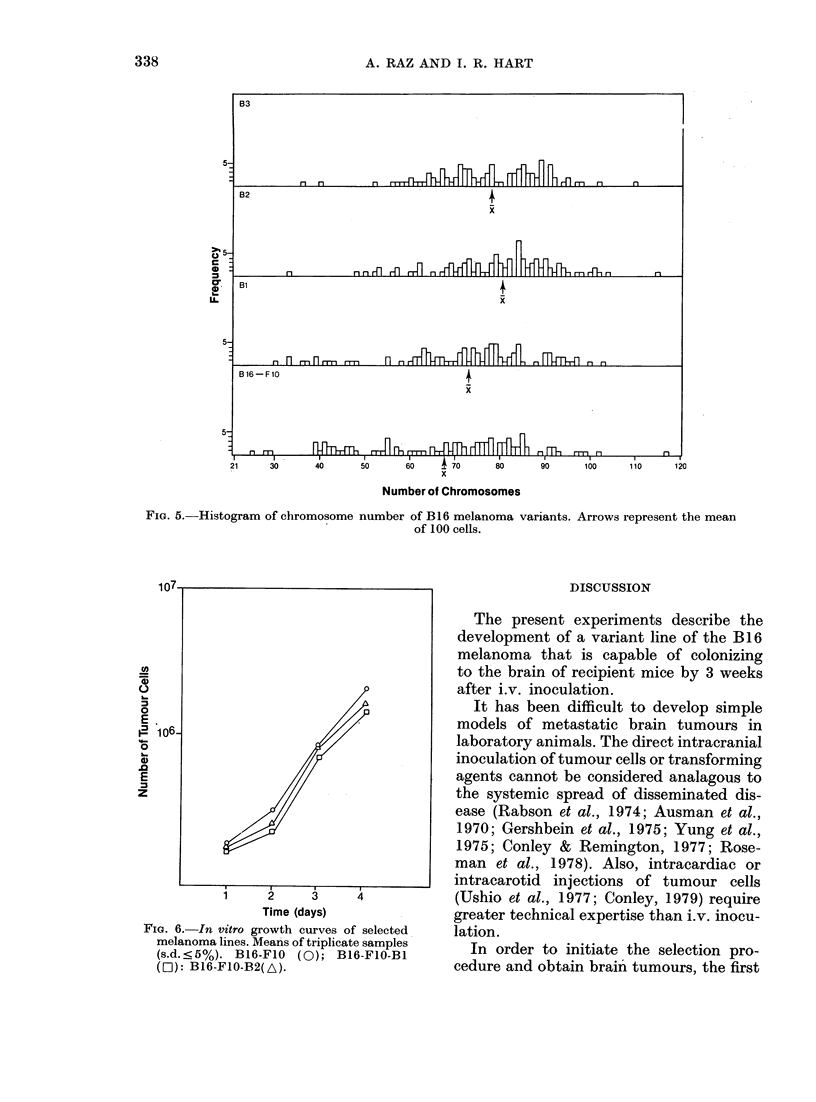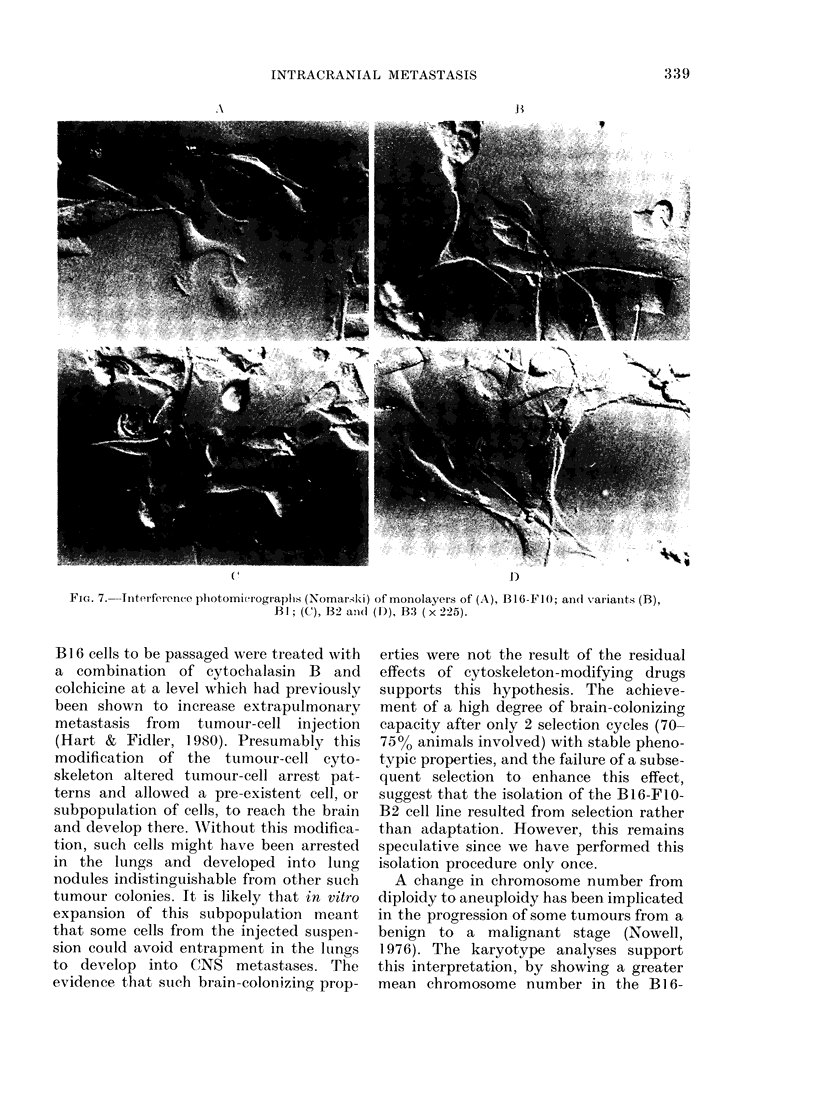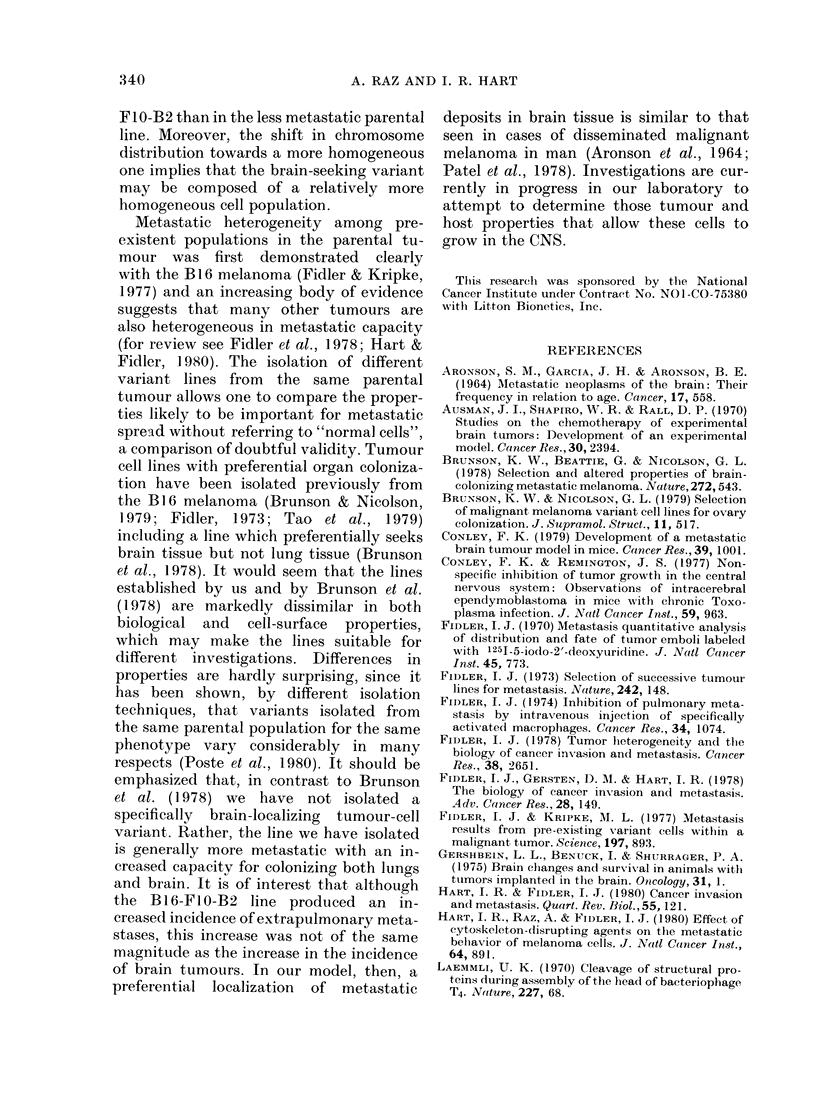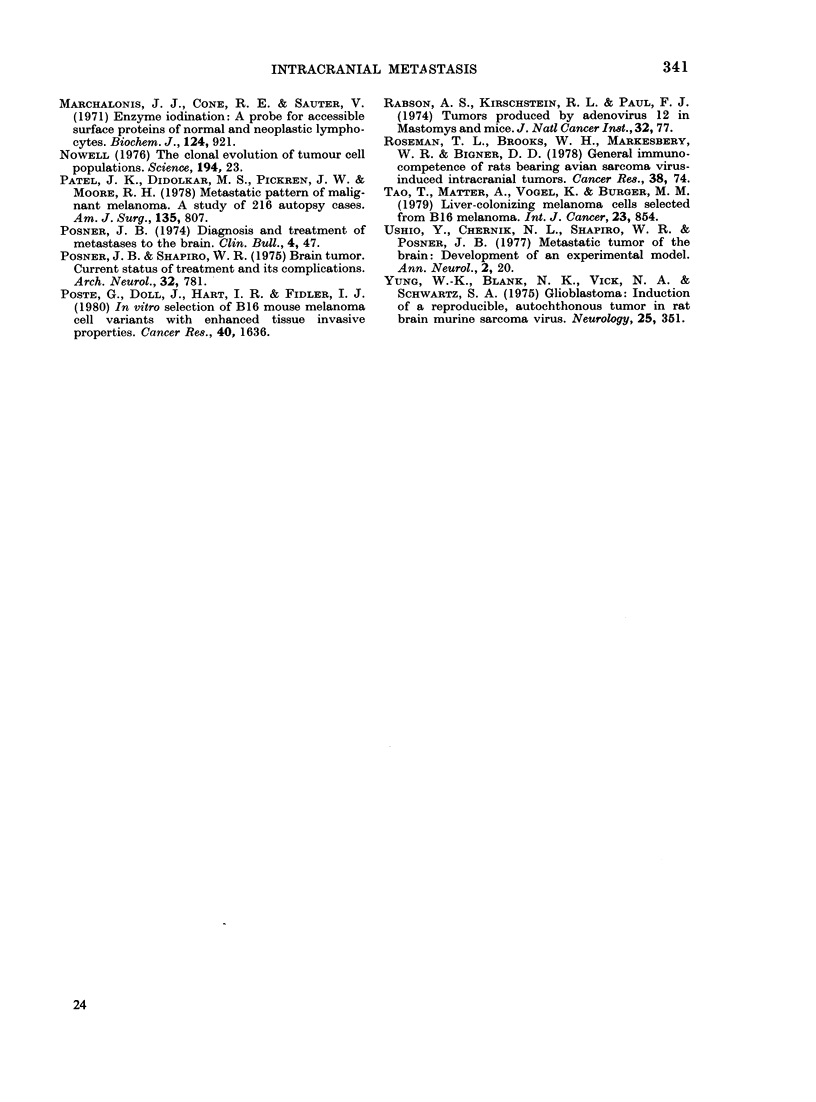# Murine melanoma: a model for intracranial metastasis.

**DOI:** 10.1038/bjc.1980.235

**Published:** 1980-08

**Authors:** A. Raz, I. R. Hart

## Abstract

**Images:**


*Br. J. Cancer* (1980) **42**, 331

### MURINE MELANOMA: A MODEL FOR INTRACRANIAL METASTASIS

A. RAZ AND I. R. HART

*From the Cancer Metastasis and Treatment Laboratory, NCI Frederick Cancer Research Center, Frederick, Maryland* 21701, *U.S.A.*

Received 7 January 1980 Accepted 6 May 1980

**Summary.—A variant subline (B16-F10-B2) selected from the B16-F10 melanoma cell line, shows greater metastatic capacity and preferential growth in the brain after i.v. injection into C57BL/6 mice. Several biological properties of these two cell lines have been compared, in an an effort to determine the mechanisms responsible for this non-random metastatic pattern. No differences in cell morphology, *in vitro* growth rates or exposed cell-surface proteins were detected. Quantitative analysis of tumour-cell arrest and distribution using $^{125}$IUdR-labelled cells indicated that, although initial arrest patterns of the cell lines were very similar, B16-F10-B2 cells survived in the lungs to a greater extent than B16-F10 cells. Karyotype analysis revealed that the B16-F10-B2 cell line had a higher mean number of chromosomes than the parent line, whereas the variance of chromosome distribution was less. We suggest that the selection of a brain-colonizing variant represents the emergence of a pre-existing subpopulation of cells, and provides a useful model for studying mechanisms of intracranial metastasis.**

INTRACRANIAL METASTASES are a frequent complication of systemic cancer. It has been reported that 16·5% of patients with generalized neoplastic disease have intracranial metastases at necropsy (Aronson *et al.*, 1964). Of patients afflicted with tumours such as malignant melanoma, the percentage affected by secondary brain tumours can be as high as 50–60% (Aronson *et al.*, 1964; Posner & Shapiro, 1975; Posner, 1974; Patel *et al.*, 1978). The effect of tumour development in the central nervous system (CNS) can be particularly devastating, since most patients develop major neurological symptoms that are both physically and psychologically debilitating (Posner & Shapiro, 1975). Survival after the development of cerebral metastases is rare, and treatment is generally unsuccessful in spite of increasingly aggressive therapeutic interventions (Posner, 1974; Posner & Shapiro, 1975).

The difficulty in developing effective therapeutic modalities for intracranial metastases is in part due to a lack of suitable experimental models of metastatic brain tumours (Conley, 1979). In determining therapeutic regimens, the value of results from experimental animal systems depends on the use of a biologically appropriate model (Fidler, 1978). There would appear then to be a need for a suitable model for intracranial metastasis.

The induction of rodent brain tumours by direct intracranial inoculation of tumour cells (Conley & Remington, 1977; Ausman *et al.*, 1970; Gershbein *et al.*, 1975) or virus particles (Rabson *et al.*, 1974; Roseman *et al.*, 1978; Yung *et al.*, 1975) circumvents the need for tumour cells to cross the blood–brain barrier, as they must do in the clinical situation. The use of intracarotid (Ushio *et al.*, 1977) or intracardiac (Conley, 1979) injections of tumour cells has produced experimental brain metastases in rodents, but these techniques require greater dexterity and technical ability than the more simple i.v. injection.

Malignant melanoma in man disseminates predominantly to both the lungs and the brain (Patel *et al.*, 1978). A brain-seeking variant line has been isolated from a murine B16 melanoma, but its propensity for the CNS appeared to be at the expense of its lung-colonizing ability (Brunson *et al*, 1978). In our laboratory we are investigating certain aspects of the biology of metastasis, using the B16 melanoma syngeneic to C57BL/6 mice, which does not grow routinely in the brains of mice after i.v. inoculation. During a recent study we observed that, if B16-F10 melanoma cells were treated with cytoskeleton-disrupting drugs before injecting them into mice, altered metastatic patterns were produced (Hart & Fidler, 1980). In the present study we report how we have used this fact to select for a highly malignant variant of the B16 melanoma that will produce both lung and brain tumours in mice after i.v. injection of tumour-cell suspensions. This cell line very closely mimics the behaviour of human tumours, and should serve as a useful animal model for the study of brain metastasis.

### MATERIALS AND METHODS

*Mice.*—Male C57BL/6 mice aged 6–8 weeks were obtained from the Animal Production Area of the Frederick Cancer Research Center.

*Cell lines.*—The original tumour line was the B16-F10 melanoma, syngeneic to the C57BL/6 mouse, selected by Fidler (1973) for its considerable ability to colonize to the lung. Tumour cells were grown in Falcon tissue-culture flasks with Eagle's minimum essential medium (EMEM) as described previously (Fidler, 1974). The tumour cell lines were tested for and found free of mycoplasma and the following murine viruses: reovirus Type 3, pneumonia virus of mice, K virus, Theiler's encephalitis virus, Sendai virus, minute virus of mice, mouse adenovirus, mouse hepatitis virus, lymphocytic choriomeningitis virus, ectromelia virus and lactate dehydrogenase virus (Microbiological Associates, Walkersville, MD).

For *in vivo* studies the tumour cells were harvested from subconfluent cultures in exponential growth by overlaying the cells with a thin layer of 0·25% trypsin–0·02% EDTA for 2 min. The cells were then washed and resuspended in Hanks' balanced salt solution (HBSS). Tumour-cell viability was assessed by trypan-blue exclusion, and only suspensions with single cells of viability >95% were used in these studies.

*Selection of brain-colonizing variant.*—B16-F10 tumour cells were harvested as described above and their number was adjusted to $2{\cdot}5 \times 10^5$ single viable cells/ml HBSS. The cell suspension was then treated with cytochalasin B (CB, Calbiochem, San Diego, CA) dissolved in dimethyl sulphoxide (5 mg/ml) and colchicine (Sigma Chemical Co., St Louis, MO) to give a final concentration of 10 $\mu$g CB/ml in $5 \times 10^{-5}$M colchicine. First the suspension was incubated at 22°C for 30 min, and then agitated with a Pasteur pipette; 0·2ml volumes ($5 \times 10^4$ cells) were injected into 5 mice *via* the tail vein. The viability of the injected cells was >95% as assessed by trypan-blue exclusion.

Three weeks after the i.v. injection, the mice were killed and necropsied. Three of the 5 mice had small, black melanotic tumours in the dorsal cerebrum. A single tumour nodule was excised in a sterile manner from one of the brains and subjected to tissue-culture conditions as described above. The tumour cell line that developed and grew from these lesions was designated B16-F10-B1. When the cells were in exponential growth they were harvested as described above, adjusted to $2{\cdot}5 \times 10^5$ cells/ml and 0·2ml volumes were injected by intracardiac (*via* the left ventricle) or by i.v. routes. Ten mice from each group were inoculated. Cells recovered from a solitary tumour nodule in the brain of one mouse 3 weeks after intracardiac injection were harvested and grown exactly as described above and the cell line was designated B16-F10-B2.

*Tumour-cell dissemination and growth after i.v. injection.*—Melanoma cells were harvested, adjusted and injected *via* the tail vein as described above. Mice were killed 21 days later and necropsied; extrapulmonary tumours were noted and, with the aid of a dissecting microscope, pulmonary nodules were counted after rinsing of the lungs with water.

*Quantitative analysis of tumour-cell arrest and survival.*—Mice were injected i.v. *via* the tail vein with tumour cells prelabelled *in vitro* with $^{125}$I-iododeoxyuridine ($^{125}$IUdR). Tumour-cell DNA was labelled by the addition of 0·2 μCi $^{125}$IUdR (sp. act. 200 mCi/mmol)/ml medium to cultures in exponential growth for 24 h. The cells were harvested, washed twice with HBSS and adjusted to $2{\cdot}5 \times 10^5$ cells/ml and 0·2ml aliquots were injected into mice.

Three representative inocula from each cell suspension were placed into vials and monitored for radioactivity in a well-type NaI crystal scintillation counter to determine activity per cell. At 10, 60, 120 min and 1 day after i.v. injection, 5 mice from each group were killed. Lungs, liver, spleen and 0·2 ml of blood were collected from each animal. The head was amputated and the lower jaw removed to eliminate the thyroid; the cranium was then treated in the same way as other organs. The organs were placed in 70% ethanol, which was replaced twice in the next 2 days to remove ethanol-soluble $^{125}$I. The residual ethanol-insoluble radioactivity was considered to be associated with tumour cells viable at the time of organ removal (Fidler, 1970).

*Karyotype.*—Karyotypes of the parent B16-F10 and the selected brain-colonizing lines were prepared by conventional techniques. The cells were accumulated in metaphase by the addition of 1 μg/ml colcemid for 3 h at 37°C. The cells were then harvested from the substrate with 0·2% trypsin and washed in phosphate-buffered saline (PBS). The cell pellet was resuspended in 5 ml 0·075M KCl for 30 min at room temperature (23°C) and the cells then fixed by the addition of an equal volume of fresh fixative (3:1 methanol: acetic acid). Fixed cells were dropped on to ice-cold wet microscope slides, dried on a warm plate and stained with Giemsa solution.

In vitro *growth curves.*—Tumour cells from each line were plated at a density of $3 \times 10^5$/60mm dish in EMEM. Cultures were incubated at 37°C in 5% $CO_2$ in an humidified atmosphere. Triplicate samples were terminated 24, 48, 72 and 96 h after plating; cells were harvested with 0·2% trypsin–0·02% EDTA and counted in a Coulter counter, Model 2B1 (Coulter Electronics Inc., Hialeah, FL).

*Light microscopy.*—Melanoma cells from the tumour lines were cultivated on glass coverslips. The cells were fixed in 2·5% glutaraldehyde in PBS for 30 min at 22°C and the coverslips were mounted on glass microscope slides before examination. A Karl Zeiss microscope equipped with polarizing optics (Nomarski) was used for photography.

Brain tissue from animals with grossly obvious brain metastases was fixed in 10% buffered formal saline for 24 h, processed and embedded in paraffin wax. Sections were cut at 5 μm and stained by Ehrlich's haematoxylin and eosin (H & E).

*Labelling of cell-surface proteins.*—Tumour cell-surface membrane proteins were radioiodinated using a lactoperoxidase-catalysed iodination technique of Marchalonis *et al.* (1971). The cell-surface radiolabelled proteins were resolved by separation using sodium dodecyl sulphate-polyacrylamide gel electrophoresis (SDS-PAGE) (Laemmli, 1970).

### RESULTS

### *Selection of a brain-colonizing variant*

The treatment of B16-F10 tumour cells with CB plus colchicine before their i.v. injection into mice, led to the formation of intracranial metastases. Cells recovered from the cerebral lesions and cultured *in vitro* were designated B16-F10-B1. Injection of $5 \times 10^4$ viable cells of this line into mice by the intracardiac or i.v. route resulted in 1/10 and 5/10 mice, respectively, developing grossly obvious melanoma colonies in the cerebrum by 3 weeks after injection. The tumour line B16-F10-B2 was developed from a lesion isolated from a single mouse afflicted with a brain tumour after intracardiac injection. After isolation, the cell line was allowed to grow *in vitro* until sufficient numbers were available, when they were frozen and stored in liquid $N_2$. All comparisons between the cell lines were then based on samples taken from these frozen stocks at the same time. The results from 3 separate experiments examining the *in vivo* behaviour of the cell lines are presented in Table I. The i.v. injection of an equal number of viable tumour cells of the lines B16-F10 and B16-F10-B2 produced two very different metastatic patterns. Of 27 mice injected with $5 \times 10^4$ B16-F10 cells, none developed brain lesions, whereas 20/27 mice injected with B16-F10-B2 cells had grossly obvious brain tumours by 3 weeks from injection. The line B16-F10-B1 occupied an inter-

TABLE I.—*Metastatic behaviour of B16 melanoma variant lines**

| | B16-F10 | Variant B16-F10-B1 | Variant B16-F10-B2 | Variant B16-F10-B3 |
|---|---|---|---|---|
| *Exp.* 1 (male mice) | | | | |
| No. lung nodules: median (range) | 39 (16–49) | 234 (43–300) | > 300 | > 300 |
| No. mice with extrapulmonary metastasis and location (other than brain) | 4/10<br>3 testicle; 2 pancreas | 4/10<br>testicle | 4/9<br>testicle | 5/9<br>testicle |
| No. mice with gross brain tumours | 0/10 | 2/9 | 7/9 | 6/9 |
| *Exp.* 2 (female mice) | | | | |
| No. lung nodules: median (range) | 49 (17–121) | | > 300 | |
| No. mice with extrapulmonary metastasis and location | 4/7<br>ovaries | | 8/8<br>ovaries | |
| No. mice with gross brain tumours | 0/7 | | 6/8 | |
| *Exp.* 3 (male mice) | | | | |
| No. lung nodules: median (range) | 65 (36–128) | > 300 | > 300 | |
| No. mice with extrapulmonary metastasis and location | 3/10<br>2 testicle; 1 pancreas<br>1 masseter muscle | 6/10<br>4 testicle<br>1 adrenal<br>1 lymph node | 8/10<br>5 testicle<br>1 adrenal; 1 kidney<br>1 pancreas | |
| No. mice with gross brain tumours | 0/10 | 4/10 | 7/10 | |

* Mice injected i.v. *via* the tail vein with $5 \times 10^4$ viable cells in 0·2 ml inoculum (HBSS) and necropsied 3 weeks later.

mediate position with regard to the number of mice developing brain tumours. Another *in vivo* passage of B16-F10-B2 cells to produce the line B16-F10-B3 did not produce a noticeable change in metastatic pattern; all further experiments used the line B16-F10-B2. In order to rule out the possibility that selection of brain-colonizing tumours was due to the residual, permanent effects of these drugs, the following control experiment was performed.

FIG. 1.—Gross appearance of CNS metastases in C57BL/6 mice 3 weeks after i.v. injection of B16-F10-B2 cells.

Cells preincubated with cytochalasin B and colchicine were maintained in tissue culture for 2 weeks. They were then harvested and injected into 10 female and 10 male mice as detailed above. None of the mice so treated developed any visible brain tumours by 3 weeks after injection. There were no differences between the number of lung nodules after injection of these cells and of an equal number of B16-F10 cells into 20 control animals.

In those mice developing brain tumours, the gross lesions were obvious as small, discrete black areas, generally 2–3 per mouse, diffusely scattered throughout the dorsal cerebrum (Fig. 1). Histological examination revealed that these black areas were composed of small foci of melanoma cells, located predominantly on the surface of the cerebral hemispheres; small foci of tumour cells were found within the white matter (Fig. 2) but metastases were not seen in either the ventricles or the choroid plexus. It appeared that initially the cells were arrested and grew in the meningeal blood vessels, and later expanded and infiltrated into the brain parenchyma (Fig. 2). The B16-F10-B2 line not only gave significantly more lung nodules

FIG. 2.—Histological section of brain (cerebrum) from syngeneic C57BL/6 mouse injected with $5 \times 10^4$ viable B16-F10-B2 cells i.v. 3 weeks previously. (A) Lesion located on dorsal surface of cerebrum with contiguous cord of tumour cells penetrating into the parenchyma. (B) Arrows indicate isolated foci of tumour cells within the brain parenchyma ($\times$ 56).

than the B16-F10 line ($P \leqslant 0{\cdot}001$ by the Mann–Whitney U test) but the morphological appearance of these tumour colonies was markedly different (Fig. 3). Nodules produced by the injection of B16-F10 cells were uniformly deep black and formed discrete lesions within the lung parenchyma. Lung colonies obtained from the B16-F10-B2 line were so numerous that they involved the whole lung, and

FIG. 3.—Representative lungs from C57BL/6 mice injected with $5 \times 10^4$ cells of (a) B16-F10 cells and (b) B16-F10-B2 cells. Differences are apparent between the number and melanin content of the tumour colonies.

were a mixture of black and pearl-grey colonies, and many of the nodules were composed of a central dark core surrounded by an opalescent grey circle (Fig. 3).

Three weeks after they were inoculated with B16-F10-B2 cells, mice generally displayed no neurological signs. However, when one group of 9 mice was allowed to live over 3 weeks, they showed a high incidence (7/9) of neurological disorders associated with the formation of brain tumours by 4 weeks after injection. These neurological signs were blindness, hindleg paralysis, ataxia and staggering, circling and incoordination.

*Stability of brain-colonizing variant*

The B16-F10-B2 cell line maintained its brain-colonizing property after 4 months of continuous tissue culture or 2 s.c. passages in mice over a 2-month period,

TABLE II.—*Distribution of* $^{125}$*IUdR-radiolabelled tumour cells in C57BL/6 mice*

| | | Time after i.v. injection | | | |
|---|---|---|---|---|---|
| | | 10 min | 60 min | 120 min | 1 day |
| (a) Lung | B16-F10 | 45,833 ± 7,457* <br> 92 ± 15% | 38,969 ± 2,086 <br> 78 ± 4% | 21,590 ± 1,514 <br> 43 ± 3% | 5,399 ± 745 <br> 11 ± 1·5% |
| | B16-F10-B2 | 43,542 ± 4,067 <br> 87 ± 8% | 41,127 ± 3,503 <br> 82 ± 7% | 32,287 ± 2,814 <br> 65 ± 6% | 16,075 ± 1,700 <br> 32 ± 3% |
| (b) Brain | B16-F10 | 289 ± 150 <br> 0·6 ± 0·3% | 477 ± 69 <br> 0·9 ± 0·1% | 296 ± 100 <br> 0·6 ± 0·2% | 174 ± 28 <br> 0·3 ± 0·06% |
| | B16-F10-B2 | 166 ± 54 <br> 0·3 ± 0·1% | 398 ± 108 <br> 0·8 ± 0·2% | 498 ± 216 <br> 1·0 ± 0·4% | 229 ± 229 <br> 0·5 ± 0·5% |

* Number of viable tumour cells remaining in organ after rinsing in ethanol. Mice were injected with $5 \times 10^4$ viable tumour cells i.v. *via* the tail vein. Five mice per sample were killed and the organs were treated as in the text; results are expressed as mean ± s.d.

when injection of 50,000 viable tumour cells produced gross brain metastases in 3/9 male mice and 5/6 female mice.

*Quantitative analysis of tumour-cell arrest and survival*

This experiment was performed twice with very similar results. Data from one experiment are presented in Table II. Tumour cells of the B16-F10-B2 line survived in the lungs to a greater extent than B16-F10 cells (32% *vs* 11% viable at 1 day) in spite of initial arrest being very similar. Very few tumour cells localized in the cranium (maximum of 1% by 2 h) but there appeared to be no obvious difference between the survival of B16-F10 and B16-F10-B2 cells at this site. There might have been a trend for B16-F10-B2 cells to survive better than B16-F10 cells in the cranium but, owing to the small numbers of cells involved and the difficulty in evaluating them, the difference was not significant.

FIG. 4.—Clearance rates of $^{125}$IUdR-labelled tumour cells from the lungs of C57BL/6 mice. Data from one of two experiments points represent means of 5 individual animals (s.d. ≤ 10%). B16-F10 (△); B16-F10-B2 (○).

The clearance of B16-F10 and B16-F10-B2 from the lungs after i.v. injection is shown in Fig. 4. The clearance of cells from the lungs is biphasic, with initial rapid clearance followed by a period of slower removal. It is of interest that the rate of B16-F10-B2 clearance is slower than that of B16-F10 cells in both phases of the curve.

*Karyotype analysis*

The number and distribution of chromosomes and the mean chromosome numbers in the cells from the B16-F10, B16-F10-B1 and B16-F10-B2 melanoma cell lines are represented in Fig. 5. The values were based on 100 counts on each cell line. The mean numbers of chromosomes were 68·9, 73·4 and 80·7 for B16-F10, B16-F10-B1 and B16-F10-B2, respectively, and the means of B16-F10-B2 and B16-F10 were significantly different ($P \leqslant 0{\cdot}001$ Student's *t* test). Moreover, the variance of chromosome distribution became smaller with increasing selection (B16-F10 386·1, B16-F10-B1 345·7, B16-F10-B2 301·8).

In vitro *growth curves*

No differences were seen among the 3 cell lines (B16-F10, B16-F10-B1 and B16-F10-B2) in their *in vitro* growth rates. All 3 lines had population-doubling times of 11–12 h (Fig. 6).

*Cell morphology*

Representative interference photomicrographs (Nomarski) are shown in Fig. 7. The 4 different tumour cell lines are well attached to the substratum and appear bipolar. No gross differences in cellular morphology could be detected among any of the cell lines.

*Cell-surface proteins*

Exposed cell-surface proteins labelled with $^{125}$I by lactoperoxidase-catalyzed iodination were separated by SDS-PAGE. No major repeatable quantitative or qualitative differences in the relative expression of a single protein were detected by our techniques.

Fig. 5.—Histogram of chromosome number of B16 melanoma variants. Arrows represent the mean of 100 cells.

Fig. 6.—*In vitro* growth curves of selected melanoma lines. Means of triplicate samples (s.d.≤5%). B16-F10 (○); B16-F10-B1 (□): B16-F10-B2(△).

DISCUSSION

The present experiments describe the development of a variant line of the B16 melanoma that is capable of colonizing to the brain of recipient mice by 3 weeks after i.v. inoculation.

It has been difficult to develop simple models of metastatic brain tumours in laboratory animals. The direct intracranial inoculation of tumour cells or transforming agents cannot be considered analagous to the systemic spread of disseminated disease (Rabson *et al.*, 1974; Ausman *et al.*, 1970; Gershbein *et al.*, 1975; Yung *et al.*, 1975; Conley & Remington, 1977; Roseman *et al.*, 1978). Also, intracardiac or intracarotid injections of tumour cells (Ushio *et al.*, 1977; Conley, 1979) require greater technical expertise than i.v. inoculation.

In order to initiate the selection procedure and obtain brain tumours, the first

FIG. 7.—Interference photomicrographs (Nomarski) of monolayers of (A), B16-F10; and variants (B), B1; (C), B2 and (D), B3 (× 225).

B16 cells to be passaged were treated with a combination of cytochalasin B and colchicine at a level which had previously been shown to increase extrapulmonary metastasis from tumour-cell injection (Hart & Fidler, 1980). Presumably this modification of the tumour-cell cytoskeleton altered tumour-cell arrest patterns and allowed a pre-existent cell, or subpopulation of cells, to reach the brain and develop there. Without this modification, such cells might have been arrested in the lungs and developed into lung nodules indistinguishable from other such tumour colonies. It is likely that *in vitro* expansion of this subpopulation meant that some cells from the injected suspension could avoid entrapment in the lungs to develop into CNS metastases. The evidence that such brain-colonizing properties were not the result of the residual effects of cytoskeleton-modifying drugs supports this hypothesis. The achievement of a high degree of brain-colonizing capacity after only 2 selection cycles (70–75% animals involved) with stable phenotypic properties, and the failure of a subsequent selection to enhance this effect, suggest that the isolation of the B16-F10-B2 cell line resulted from selection rather than adaptation. However, this remains speculative since we have performed this isolation procedure only once.

A change in chromosome number from diploidy to aneuploidy has been implicated in the progression of some tumours from a benign to a malignant stage (Nowell, 1976). The karyotype analyses support this interpretation, by showing a greater mean chromosome number in the B16-

F10-B2 than in the less metastatic parental line. Moreover, the shift in chromosome distribution towards a more homogeneous one implies that the brain-seeking variant may be composed of a relatively more homogeneous cell population.

Metastatic heterogeneity among pre-existent populations in the parental tumour was first demonstrated clearly with the B16 melanoma (Fidler & Kripke, 1977) and an increasing body of evidence suggests that many other tumours are also heterogeneous in metastatic capacity (for review see Fidler *et al.*, 1978; Hart & Fidler, 1980). The isolation of different variant lines from the same parental tumour allows one to compare the properties likely to be important for metastatic spread without referring to "normal cells", a comparison of doubtful validity. Tumour cell lines with preferential organ colonization have been isolated previously from the B16 melanoma (Brunson & Nicolson, 1979; Fidler, 1973; Tao *et al.*, 1979) including a line which preferentially seeks brain tissue but not lung tissue (Brunson *et al.*, 1978). It would seem that the lines established by us and by Brunson *et al.* (1978) are markedly dissimilar in both biological and cell-surface properties, which may make the lines suitable for different investigations. Differences in properties are hardly surprising, since it has been shown, by different isolation techniques, that variants isolated from the same parental population for the same phenotype vary considerably in many respects (Poste *et al.*, 1980). It should be emphasized that, in contrast to Brunson *et al.* (1978) we have not isolated a specifically brain-localizing tumour-cell variant. Rather, the line we have isolated is generally more metastatic with an increased capacity for colonizing both lungs and brain. It is of interest that although the B16-F10-B2 line produced an increased incidence of extrapulmonary metastases, this increase was not of the same magnitude as the increase in the incidence of brain tumours. In our model, then, a preferential localization of metastatic deposits in brain tissue is similar to that seen in cases of disseminated malignant melanoma in man (Aronson *et al.*, 1964; Patel *et al.*, 1978). Investigations are currently in progress in our laboratory to attempt to determine those tumour and host properties that allow these cells to grow in the CNS.

This research was sponsored by the National Cancer Institute under Contract No. NO1-CO-75380 with Litton Bionetics, Inc.

### REFERENCES

Aronson, S. M., Garcia, J. H. & Aronson, B. E. (1964) Metastatic neoplasms of the brain: Their frequency in relation to age. *Cancer*, **17**, 558.

Ausman, J. I., Shapiro, W. R. & Rall, D. P. (1970) Studies on the chemotherapy of experimental brain tumors: Development of an experimental model. *Cancer Res.*, **30**, 2394.

Brunson, K. W., Beattie, G. & Nicolson, G. L. (1978) Selection and altered properties of brain-colonizing metastatic melanoma. *Nature*, **272**, 543.

Brunson, K. W. & Nicolson, G. L. (1979) Selection of malignant melanoma variant cell lines for ovary colonization. *J. Supramol. Struct.*, **11**, 517.

Conley, F. K. (1979) Development of a metastatic brain tumour model in mice. *Cancer Res.*, **39**, 1001.

Conley, F. K. & Remington, J. S. (1977) Non-specific inhibition of tumor growth in the central nervous system: Observations of intracerebral ependymoblastoma in mice with chronic Toxoplasma infection. *J. Natl Cancer Inst.*, **59**, 963.

Fidler, I. J. (1970) Metastasis quantitative analysis of distribution and fate of tumor emboli labeled with $^{125}$I-5-iodo-2′-deoxyuridine. *J. Natl Cancer Inst.* **45**, 773.

Fidler, I. J. (1973) Selection of successive tumour lines for metastasis. *Nature*, **242**, 148.

Fidler, I. J. (1974) Inhibition of pulmonary metastasis by intravenous injection of specifically activated macrophages. *Cancer Res.*, **34**, 1074.

Fidler, I. J. (1978) Tumor heterogeneity and the biology of cancer invasion and metastasis. *Cancer Res.*, **38**, 2651.

Fidler, I. J., Gersten, D. M. & Hart, I. R. (1978) The biology of cancer invasion and metastasis. *Adv. Cancer Res.*, **28**, 149.

Fidler, I. J. & Kripke, M. L. (1977) Metastasis results from pre-existing variant cells within a malignant tumor. *Science*, **197**, 893.

Gershbein, L. L., Benuck, I. & Shurrager, P. A. (1975) Brain changes and survival in animals with tumors implanted in the brain. *Oncology*, **31**, 1.

Hart, I. R. & Fidler, I. J. (1980) Cancer invasion and metastasis. *Quart. Rev. Biol.*, **55**, 121.

Hart, I. R., Raz, A. & Fidler, I. J. (1980) Effect of cytoskeleton-disrupting agents on the metastatic behavior of melanoma cells. *J. Natl Cancer Inst.*, **64**, 891.

Laemmli, U. K. (1970) Cleavage of structural proteins during assembly of the head of bacteriophage $T_4$. *Nature*, **227**, 68.

Marchalonis, J. J., Cone, R. E. & Sauter, V. (1971) Enzyme iodination: A probe for accessible surface proteins of normal and neoplastic lymphocytes. *Biochem. J.*, **124**, 921.

Nowell (1976) The clonal evolution of tumour cell populations. *Science*, **194**, 23.

Patel, J. K., Didolkar, M. S., Pickren, J. W. & Moore, R. H. (1978) Metastatic pattern of malignant melanoma. A study of 216 autopsy cases. *Am. J. Surg.*, **135**, 807.

Posner, J. B. (1974) Diagnosis and treatment of metastases to the brain. *Clin. Bull.*, **4**, 47.

Posner, J. B. & Shapiro, W. R. (1975) Brain tumor. Current status of treatment and its complications. *Arch. Neurol.*, **32**, 781.

Poste, G., Doll, J., Hart, I. R. & Fidler, I. J. (1980) *In vitro* selection of B16 mouse melanoma cell variants with enhanced tissue invasive properties. *Cancer Res.*, **40**, 1636.

Rabson, A. S., Kirschstein, R. L. & Paul, F. J. (1974) Tumors produced by adenovirus 12 in Mastomys and mice. *J. Natl Cancer Inst.*, **32**, 77.

Roseman, T. L., Brooks, W. H., Markesbery, W. R. & Bigner, D. D. (1978) General immunocompetence of rats bearing avian sarcoma virus-induced intracranial tumors. *Cancer Res.*, **38**, 74.

Tao, T., Matter, A., Vogel, K. & Burger, M. M. (1979) Liver-colonizing melanoma cells selected from B16 melanoma. *Int. J. Cancer*, **23**, 854.

Ushio, Y., Chernik, N. L., Shapiro, W. R. & Posner, J. B. (1977) Metastatic tumor of the brain: Development of an experimental model. *Ann. Neurol.*, **2**, 20.

Yung, W.-K., Blank, N. K., Vick, N. A. & Schwartz, S. A. (1975) Glioblastoma: Induction of a reproducible, autochthonous tumor in rat brain murine sarcoma virus. *Neurology*, **25**, 351.